# Single Immunization of a Vaccine Vectored by a Novel Recombinant Vaccinia Virus Affords Effective Protection Against Respiratory Syncytial Virus Infection in Cotton Rats

**DOI:** 10.3389/fimmu.2021.747866

**Published:** 2021-09-17

**Authors:** Marsha S. Russell, Sathya N. Thulasi Raman, Caroline Gravel, Wanyue Zhang, Annabelle Pfeifle, Wangxue Chen, Gary Van Domselaar, David Safronetz, Michael Johnston, Simon Sauve, Lisheng Wang, Michael Rosu-Myles, Jingxin Cao, Xuguang Li

**Affiliations:** ^1^Centre for Biologics Evaluation, Biologic and Radiopharmaceutical Drugs Directorate, Health Products and Food Branch (HPFB), Health Canada and WHO Collaborating Center for Standardization and Evaluation of Biologicals, Ottawa, ON, Canada; ^2^Department of Biochemistry, Microbiology and Immunology, Faculty of Medicine, University of Ottawa, Ottawa, ON, Canada; ^3^National Research Council of Canada, Human Health Therapeutics, Ottawa, ON, Canada; ^4^National Microbiology Laboratory, Public Health Agency of Canada, Winnipeg, MB, Canada; ^5^Department of Chemistry, Carleton University, Ottawa, ON, Canada

**Keywords:** vaccine, antibodies, vaccinia, virus, vector, respiratory syncytial virus

## Abstract

Respiratory syncytial virus (RSV) is a leading cause of respiratory infections worldwide and disease management measures are hampered by the lack of a safe and effective vaccine against the infection. We constructed a novel recombinant RSV vaccine candidate based on a deletion mutant vaccinia virus platform, in that the host range genes E3L and K3L were deleted (designated as VACVΔE3LΔK3L) and a poxvirus K3L ortholog gene was used as a marker for the rapid and efficient selection of recombinant viruses. The safety of the modified vaccinia virus was investigated by intranasal administration of BALB/c mice with the modified vaccinia vector using a dose known to be lethal in the wild-type Western Reserve. Only a minor loss of body weight by less than 5% and mild pulmonary inflammation were observed, both of which were transient in nature following nasal administration of the high-dose modified vaccinia virus. In addition, the viruses were cleared from the lung in 2 days with no viral invasions of the brain and other vital organs. These results suggest that the virulence of the virus has been essentially abolished. We then investigated the efficiency of the vector for the delivery of vaccines against RSV through comparison with another RSV vaccine delivered by the widely used Modified Vaccinia virus Ankara (MVA) backbone. In the cotton rats, we found a single intramuscular administration of VACVΔE3LΔK3L-vectored vaccine elicited immune responses and protection at a level comparable to the MVA-vectored vaccine against RSV infection. The distinct features of this novel VACV vector, such as an E3L deletion for attenuation and a K3L ortholog for positive selection and high efficiency for vaccine delivery, could provide unique advantages to the application of VACV as a platform for vaccine development.

## Introduction

Respiratory syncytial virus (RSV) infections are the most common cause of severe respiratory disease in infants and was attributable to 33.1 million cases of Acute Lower respiratory tract infections worldwide in 2015 (1). Even though the burden of RSV disease on the world population is well documented, there is still a lack of safe and effective vaccines against this disease. The absence of a safe and effective prophylactic treatment, in spite of decades of research, necessitates studies on the understanding and characterization of different platforms that can be used for RSV vaccine development. One of the promising platforms is Vaccinia virus (VACV) vector backbone for delivery of foreign antigens. Their large capacity for packaging heterologous DNA and wide usage in smallpox vaccination campaigns have established them as an ideal candidate for the construction of vaccines against a wide variety of pathogens (2).

While VACV has a long history of usage in the smallpox eradication campaign, it is not without serious adverse events. Smallpox vaccination has been recorded to cause serious adverse events such as post vaccinial central nervous system disease, progressive vaccinia, fetal vaccinia and Eczema vaccinatum (3–5). As a result, the first generation of VACV strains used in the smallpox vaccination campaign are not suitable for contemporary vaccine development and several attenuated strains have been developed for this purpose (6). One of the well-researched strain used for the purpose of vaccine design is Modified Vaccinia virus Ankara (MVA), which is a non-replicating VACV that was attenuated by passaging over 500 times in Chicken Embryo Fibroblasts (CEF) (7). Because of successive repeated passaging in avian cells the MVA strain lost approximately 15% of its genome and thereby does not replicate in human cells (8). Many of the modern studies on VACV-vectored cancer and infectious disease vaccines utilize the MVA strain as the backbone due to its non-replicating nature and improved safety profile compared to early VACV strains (2, 9, 10). However, non-replicating poxviruses such as MVA are not as immunogenic or protective as replicating poxviruses in protecting against an Orthopoxvirus challenge (11). Additionally, replicating poxviruses afford better protection and immunogenicity as vaccine vectors, when compared to non-replicating viruses (12–14). Therefore, different replicating and non-replicating vaccinia virus based platforms are valuable for vaccine development and research.

Previously it has been shown that deletion of the host range gene E3L from vaccinia virus would render the virus highly attenuated and also more potent at inducing innate immune responses, such as type-1 interferon response (15–18). More recently, we have described a fast, highly efficient and cost-effective method to construct recombinant VACV by combining the deletion of the host range gene E3L and swap of another VACV host range gene K3L with a poxvirus K3L ortholog gene (19). Since the E3L deletion mutant VACV is more potent at inducing innate immune responses and is highly attenuated, this platform has the potential to be used for the generation of recombinant vaccines, in particular against the pathogens to which efficacious vaccines remain elusive, such as RSV.

The RSV genome encodes 11 proteins, 3 of which are displayed on the viral envelope namely the small hydrophobic (SH) protein, the Major surface glycorprotein G and the Fusion glycoprotein F (20). While both F and G proteins can elicit neutralizing antibodies, the majority of the vaccines currently under clinical development target the F protein (21). The reasons for this preference include the following: 1) F protein is essential for virus entry (22); 2) Unlike the G protein, F protein is highly conserved among different strains of RSV (22); 3) it is well established that a monoclonal antibody directed against the F protein (palivizumab) can reduce severe disease in high-risk infants (23, 24).

In this communication, we report the development of a novel experimental RSV vaccine encoding the RSV-F gene, delivered using a modified Western reserve (WR) strain with E3L and K3L deletions (WR-RSVF). The immune responses and the protective efficacy of the WR-RSVF was evaluated in a cotton rat model of RSV infection.

## Materials and Methods

### Animals and Ethics Statement

Six to seven week old female cotton rats were obtained from Envigo, Somerset, N.J., USA. Six to eight week old female Balb/c mice were obtained from Charles River, Senneville, Quebec, Canada. All animal procedures were approved by the Animal Care Committee in Health Canada, Ottawa and performed in accordance with institutional guidelines.

### Cells and Viruses

RSV A2 strain (ATCC VR-1540, Manassas, Va) was propagated in HEp-2 cells (ATCC CCL-23, Manassas, Va) cultivated in growth media as previously described (25). Briefly, after absorption with a multiplicity of 0.02, cells were cultured in serum-free medium until 80% cytopathic effect was reached. Culture media and cells were harvested, lysed by two cycles of freeze/thaw, centrifuged, and filtered. The resulting supernatant was centrifuged at 62,000 ×g for 30 min at 4°C on a 30% sucrose cushion and the resulting pellet was resuspended in HBSS with 25 mM HEPES buffer.

Dr. Bernard Moss at the National Institutes of Health kindly provided recombinant MVA viruses expressing the RSV F protein and ‘Empty’ virus (26). The virus stock was prepared on chicken embryo fibroblasts (CEF) cells and purified on a sucrose cushion as described previously (27). The resulting virus was immune-titrated on DF-1 cells with Rabbit Anti-Vaccinia Virus Polyclonal Ab from Thermo (Cat# PA17258) as the primary antibody followed by the secondary antibody, ECL Donkey Anti-Rabbit HRP (Amersham Cat#NA934V) and DAB substrate detection.

The VACVΔE3LΔK3L based on the Western Reserve strain (WR) was described previously (19) and was propagated in HeLaPKRko cells cultivated in growth media (Dulbecco’s Modified Eagle Medium supplemented with 1.5 g/L sodium bicarbonate, 2 mM Glutamax, 25 mM HEPES, 20 U/ml Penicillin, 0.02 mg/ml Streptomycin, and 10% FBS). The recombined progeny virus containing the gene of interest are selected and propagated on BHK21 cells, which are cultivated in the same growth media as the parental strain.

RK13 cells were used for viral titration of recombinant VACV virus. RK13 cell-line was obtained from ATCC (ATCC CCL-37, Manassas, Va) and cultivated in the same growth media as the HeLaPKRko cells above.

### Generation of Recombinant Vaccinia Virus

The full length of RSV-A2 F gene (GenBank accession #KJ155694.1) as a secreted form with the inclusion of 23 amino acids from the human tyrosinase signal peptide (MLLAVLYCLLWSFQTSAGHFPRA; GenBank accession #AH003020) at the N-terminus (VV-WR-RSVF) driven by VACV mH5 promoter (28) was synthesized by BioBasic (Toronto, Canada). This RSVF gene was fused between the right and left flanking regions containing the poxvirus K3L ortholog taterapox virus 037 (TATV037) using overlapping PCR with Phusion High-Fidelity DNA Polymerase (Life Technologies) and the following primers - 5’-TACGCTACTATACCGGCATT-3’, 5’-GGATCAGCATCTGGTACAAT-3’. A corresponding Empty viral vector backbone was also included (VV-WR-Empty).

Recombinant VACV was generated as previously described (19). Briefly, the recombinant shuttle vector DNA was transfected (Attractene, QIAGEN) into the parental virus (VACVΔE3LΔK3L)-infected HeLa/PKRko cells. Following 48 hours of incubation, the virus was harvested and passaged twice in BHK21 cells. Since the parental VACVΔE3LΔK3L carries EGFP gene and the mCherry gene in the E3L and K3L locus respectively; and insertion site for the RSVF gene was the K3L locus, the stable recombinant virus was plaque purified by selecting plaques expressing only EGFP.

The selected recombinant VACV expressing RSVF was amplified in BHK21 cells, partially purified through 35% sucrose cushion, and the titre was determined on RK13 cells using standard plaque assay. For titration, serial dilutions of the virus were incubated on RK13 cells for 2 hours at 37°C. A 1:1 overlay of 2× DMEM media with 4% FBS and 0.8% agarose was added. Following 3 days of incubation, the overlay was removed and the cell monolayer was stained with crystal violet before counting plaques.

### Immunization and Infection

For challenge studies, on day 0, VV-WR-RSVF, VV-WR-Empty, VV-MVA-RSVF, VV-MVA-Empty at 1 × 10^7^ PFU or PBS as an additional control were injected intramuscularly into the cotton rats at a volume of 100µl, with 5 cotton rats per treatment group. On day 21, all animals were challenged intranasally with RSV-A2 at 1 × 10^6^ PFU. Five days post-challenge, the animals were sacrificed (**Figure 3B**). Lungs from the same animal were used for viral titration as well as histology. To accomplish this, the right lobe was used for virus titration and the left lobe was fixed in 10% neutral buffered formalin (Sigma) under 25 cm of water pressure. The blood was obtained *via* abdominal aorta bleed. The nose was aseptically removed into 300µl HEp-2 growth media and snap frozen in liquid N2 for viral load determination.

**Figure 1:**
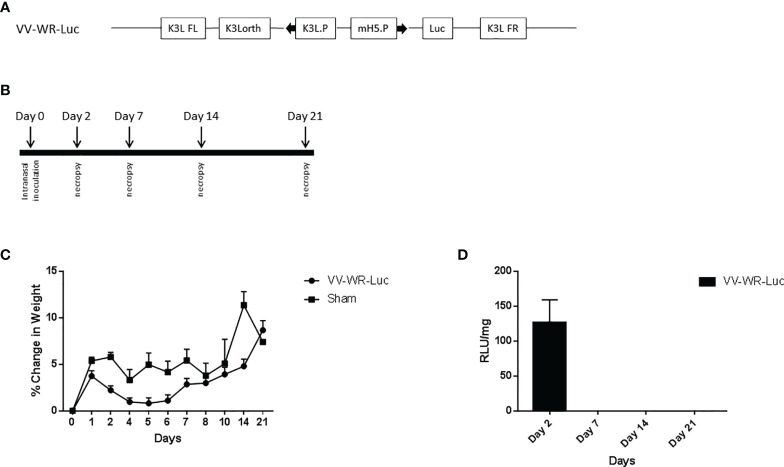
Vaccinia vectored construct and intranasal inoculation regimen for virulence assessment. **(A)** Schematic representation of the WR vaccinia virus construct VV-WR-Luc expressing the firefly luciferase gene. The luciferase protein transgene is under the control of the mH5 promoter, mH5.P. This sequence is flanked by the K3L ortholog regions. **(B)** Schematic diagram of the intranasal inoculation and necropsy timeline. **(C, D)** Intranasal administration of VV-WR-Luc results in minor transient weight loss and localized transgene expression. Balb/c mice were intranasally administered 10^6^ PFU of the VV-WR-Luc construct or sham and daily bodyweights were measured. Mice were necropsied at day 2, day 7, day, 14, and day 21 with n = 5 mice per timepoint per group. **(C)** % change in body weight compared to day 0 and **(D)** Transgene expression in the lungs expressed as Relative Luminescence Units (RLU)/mg of tissue is shown. Brain and trachea not shown.

For safety assessment, on day 0, VV-WR-Luc, was administered intranasally into Balb/c mice at 1 × 10^6^ PFU in 25µl volume, divided evenly between nares. On day 2, day 7, day 14, and day 21, mice were sacrificed (**Figure 1B**) at 5 animals per time point and the lungs, trachea, and brain were collected. The lungs from the same animal were used for transgene expression evaluation as well as histopathology assessment. To accomplish this, the right lobe was used for transgene expression evaluation and the left lobe was fixed in 10% neutral buffered formalin (Sigma) under 25 cm of water pressure. The right lung lobe, trachea, and brain were snap frozen in liquid N2 for transgene expression determination.

### Histopathology

For the challenge study in cotton rats, five days post RSV challenge, the left lobe of the lung was collected as described above and fixed in 10% neutral buffered formalin. Four transverse sections at different levels of the lobe were trimmed, processed, embedded into paraffin blocks, and microtomed to produce four-micron sections, which were stained with Hematoxylin and Eosin (H&E) for evaluation. Scoring was done by a certified veterinary pathologist who was blinded to the experimental design. A numeric scale (0-5) was employed to grade lung lesion severity where 0 means lesion not present; 1 means minimal; 2 means mild; 3 means moderate; 4 means marked; and 5 means severe. Total pathological score was calculated as the average of the individual scores. Bronchiolar epithelial hyperplasia denotes thickening of the epithelium by multiple layers of cells and follows loss of the epithelium.

For the safety assessment in mice, the same procedure as above was used at each necropsy time point outlined in **Figure 1B**. Samples were processed and scored in a similar manner as well.

### Lung and Nose Viral Titration

Tissue for lung viral titration was obtained as described above and performed as previously described (25). Briefly, half the lung was weighed, homogenized and serial dilutions were performed on HEp-2 cells in 6-well plates for 2 h at 37°C. The virus inoculum was removed and the monolayer was overlaid with a 1:1 mixture of HEp-2 growth medium and 0.8% SeaKem ME agarose (Lonza Rockland ME). After incubation for 7 days at 37°C, 5% CO_2_, plates were stained with crystal violet (Sigma-Aldrich) and plaques were quantified.

Tissue for nose viral titration was obtained as described above. The nasal tissue was homogenized using a chilled tissue grinder in the HEp-2 growth media it was frozen in and then plaque assayed as above.

### Enzyme Linked Immunosorbent Assay (ELISA)

Serum from immunized and challenged cotton rats were collected for determination of IgG levels as previously described (25) with modifications. Ninety six-well plates were coated with 0.5ug/ml recombinant F protein (Sino #11049-V08B) overnight at 4°C in carbonate buffer. Next day, the plates were washed and blocked with 3% BSA in PBS containing 0.05% Tween 20 for 1 h at 37°C. Serial dilutions of the cotton rat serum in blocking buffer were then added for 1 h at 37°C. After washing, HRP-conjugated goat anti-mouse IgG HRP (Southern Biotech Cat#1030-05) diluted in blocking buffer at 1:5,000 was added for 1 h at 37°C. Following a subsequent wash, Tetramethylbenzidine substrate (Cell Signaling Technology) was added and the reaction was then stopped with 0.16 M sulfuric acid. The plates were read spectrophotometrically at 450 nm.

### RSV Microneutralization and ADCC Assay

RSV-neutralizing ability of the serum from immunized and challenged Cotton Rats was determined as previously described (25, 29). Briefly, serial dilutions of the serum were incubated with RSV-A2 for 1 hour at 37°C and 5% CO2. The diluted serum were added to HEp-2 cells, which were seeded the previous day and incubated at 37°C. Three days later, the cells were fixed with ice-cold methanol and blocked with 5% non-fat dry milk in PBS containing 0.1% Tween 20 for 2 hours at 37°C. Then, the plates were washed and a HRP-conjugated anti-RSV antibody (Meridian Life Science) was added for 1 hour at 37°C. After a subsequent wash, Tetramethylbenzidine substrate (Cell Signaling Technology) was added and the reaction was stopped with 0.16 M sulfuric acid. The plates were read spectrophotometrically at 450 nm.

The ADCC activities of the serum Abs were measured with the Promega ADCC Reporter Bioassay (Promega, G7102, Madison, WI, USA) according to the manufacturer’s instructions. It has been shown that the mouse FcγRIV receptor is closely related to hamster FcγRIV receptor (30). Given that hamsters and cotton rats are within the Cricetidae family, we postulated that cotton rat serum antibodies would be able to bind and activate the FcγRIV receptor on the mouse reporter cells used in this bioassay.

Briefly, 20,000 HEp-2 cells were seeded in a white clear bottom 96-well plate. The next day, the cells were infected with RSV-A2 at a multiplicity of 5 and incubated for 15-20 hours at 37°C, 5% CO2. The following day, the serum was heat-inactivated at 56°C for 30min and serially diluted in a 96-well round bottom plate. The reporter cells were then added to the diluted serum at 100,000 cells/well. The media was removed from the infected HEp-2 cells and the serum/reporter cell mixture was added to the RSV infected HEp-2 cells. This was incubated for 4 hours and 45 minutes at 37°C, 5% CO2 at which time the plate was moved to room temperature for 15 minutes to acclimate before adding the Bio-Glo luciferase buffer. After 2 minutes, the luminescence reading was measured.

### Quantification of Transgene Expression in Organs

Transgene expression was evaluated in the lungs, trachea, and brain of mice. 1ml of GLO lysis buffer (Promega E2661) was added for every 50mg tissue to organs that were snap frozen at necropsy. After homogenization, and incubation at room temperature for 10 minutes, lysates were frozen again for downstream processing. Frozen lysates were thawed and clarified by centrifugation. Cleared lysates were then mixed 1:1 with GLO lysis reagent in white plates. Luminescence was read after 5 mins and expressed as Relative Luciferase Units (RLU)/mg of tissue.

### Statistical Analysis

Analysis was conducted using non-parametric unpaired t-test where appropriate. Tests were done at a 5% significance level. All statistical analyses were performed using GraphPad Prism 7 software.

## Results

### Novel Vaccinia WR Backbone Is Avirulent in Mice

**Figure 2:**
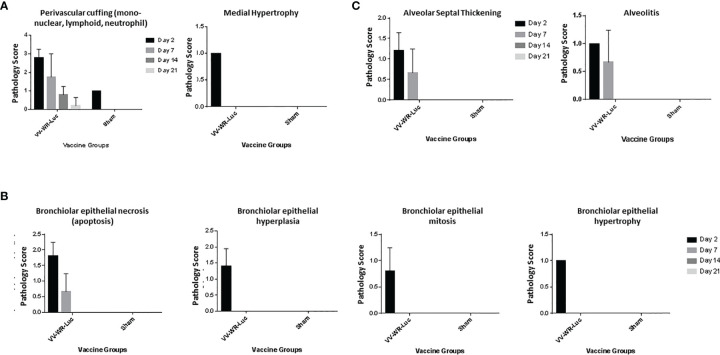
Intranasal administration of VV-WR-Luc causes transient and mild pathology in the lungs. Balb/c mice were intranasally administered 10^6^ PFU of the VV-WR-Luc construct or sham and necropsied at day 2, day 7, day, 14, and day 21 with n = 5 mice per time point per group. The left lobe of the lung was evaluated for histopathological changes. The Pathological scoring of the vasculature **(A)**, bronchiolar **(B)**, and alveolar **(C)** compartments of the lungs are shown.

We first investigated the safety and biodistribution of the modified virus. To this end, the attenuation or abolishing of the virulence of the virus were evaluated in BALB/c mice using a dose that is known to be lethal to the animals when wild type (unmodified Western Reserve strain, WT-WR) is injected intranasally. Specifically, intranasal infection of Wild Type (WT) WR virus is highly lethal with a 50% lethal dose of 10^4.5^ pfu/mouse in Balb/c mice (31–34). It causes severe acute disease with high replication titers in the lungs resulting in systemic spread of the virus to various organs including the brain. Additionally, WT-WR virus also causes severe weight loss and pathology in lungs. Therefore, we first assessed the virulence of the novel WR Vaccinia virus backbone used in this study by assessing body weight loss, lung histopathology and bio-distribution in Balb/c mice. For this purpose, a VACVΔE3L/TATV037 virus expressing a luciferase gene was constructed (VV-WR-Luc) (**Figure 1A**). The mice were inoculated intranasally with VV-WR-Luc virus at a dosage of 10^6^ PFU/mouse, a dose that is normally lethal with WT-WR *via* intranasal administration. Body weight was measured every day until 21 days post infection (dpi) and groups of mice were necropsied at days 2, 7, 14 and 21 post infection in order to assess lung pathology and tissue distribution (**Figure 1B**). The mice experienced minor transient weight loss of less than 5% and recovered quickly after 5 dpi attaining a weight similar to the control uninfected mice on day 21 (**Figure 1C**). Next, to assess the bio-distribution of the recombinant WR vector in the target organs of the lungs, trachea and brain, we assessed the expression levels of luciferase in these organs from mice necropsied at various time points (**Figure 1D**). Luciferase transgene expression was transiently observed on day 2 post infection in the lungs, and was not detected thereafter at later time points. Additionally, no luciferase expression was observed in the brain and trachea at any of the days tested. Histopathological analysis of lungs showed moderate to minimal severity in the vasculature (**Figure 2A**), mild to minimal severity in the bronchioles (**Figure 2B**) and minimal severity in the alveoli (**Figure 2C**) on 2 days post infection at the 10^6^ PFU dosage. The pathological severity was highest on day 2 post infection and steadily decreased thereafter. Notably, the lung pathology was similar to the uninfected control on days 14 and 21. While a standard full toxicological assessment was not conducted, these preliminary results suggest that the modified WR-VACV is substantially attenuated.

### WR-RSVF Induced Robust Antibody Responses in Cotton Rats

**Figure 3:**
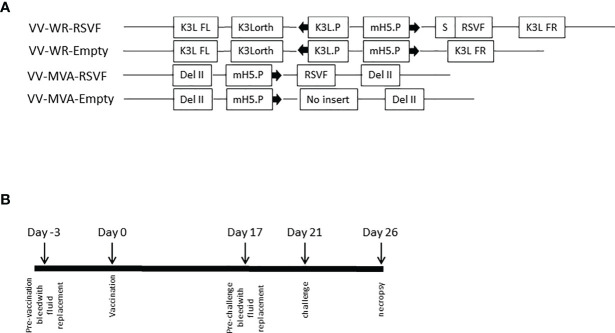
Vaccinia vectored constructs and vaccination regimen. **(A)** Schematic representation of the WR and MVA vaccinia virus constructs. Both VV-WR-RSVF and VV-MVA-RSVF express the full length RSV F protein. The VV-WR-RSVF F protein transgene is preceded by a secretion signal, S and is under the control of the mH5 promoter, mH5.P. This sequence is flanked by the K3L ortholog regions. The VV-MVA-RSVF contains the RSV F gene under the control of mH5.P promoter inserted by homologous recombination in the del II region of VACV genome, as described by Wyatt et al. (26). VV-WR-Empty and VV-MVA-Empty do not encode for RSV F **(B)** Schematic diagram of the immunization, RSV challenge and necropsy timeline.

We first assessed the WR-RSVF-induced RSV-F-specific antibody responses. To this end, four recombinant VACV were used for this purpose: VACVΔE3L/TATV037 (VV-WR-Empty), VACVΔE3L/TATV037/RSVF (VV-WR-RSVF), MVA (VV-MVA-Empty), and MVA/RSVF (VV-MVA-RSVF) (**Figure 3A**). As described in **Figure 3B**, the protection study in cotton rats involved a single intramuscular vaccination regimen, with intranasal challenge of RSV-A2 virus 3 weeks after vaccination and necropsy for tissue collection performed 5 days post viral challenge.

**Figure 4:**
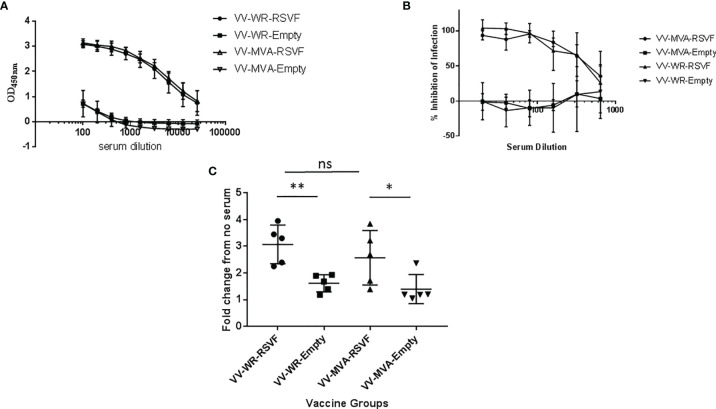
VV-WR-RSVF induces robust humoral response to RSV-A2 challenge in Cotton Rats. Cotton rats were intramuscularly immunized with one dose of 10^7^ PFU of the indicated VV-WR or VV-MVA constructs and intranasally challenged with 10^6^ PFU of RSV-A2 and necropsied 5 days later with n = 5 cotton rats per treatment group. Serum was collected and **(A)** F-specific total IgG in the serum of vaccinated cotton rats 5 days post-challenge was determined using ELISA (n = 5). **(B)** RSV neutralizing ability and **(C)** RSV Antibody-dependent cell-mediated cytotoxicity (ADCC) ability of the cotton rat serum collected 5 days post-challenge (n = 5 each) were also evaluated. *p < 0.05, **p < 0.01 (non-parametric t-test Mann-Whitney). ns, not significant.

Antibody-mediated immune responses play a vital role in protection against RSV infection (35, 36). Therefore, we first evaluated antibody responses using serum collected at necropsy, 5 days post challenge with RSV-A2. As shown in **Figure 4A**, both WR-RSVF and MVA-RSVF vaccines induced similar levels of robust RSV-F specific total IgG in the serum of vaccinated animals, when compared to WR-Empty and MVA-Empty vaccine vectors. Additionally, the levels of neutralizing antibodies elicited by vaccination and challenge were also similar in the case of both the RSVF vaccines, while the antibodies from the sera of animals vaccinated with vector alone had no detectable neutralizing activity (**Figure 4B**). Moreover, as antibody dependent cellular cytotoxicity (ADCC) is a critical component of the immune response during viral infections and RSV-specific Immunoglobulin (Ig) G antibodies have been shown to induce ADCC on RSV-infected cells (37, 38), we determined the ADCC activity induced by our vaccines. Since Fcγ receptors (FcγR) are the predominant receptors mediating ADCC, we utilized a bio-luminescent reporter assay to assess the FcγR binding ability of serum antibodies generated in response to vaccination and challenge. As shown in **Figure 4C**, serum antibodies elicited from both WR and MVA VACV vectored RSVF vaccines demonstrated comparable ADCC activity at a level significantly higher than the empty vector group.

### WR-RSVF Induced Effective Protection Against RSV Infection

**Figure 5:**
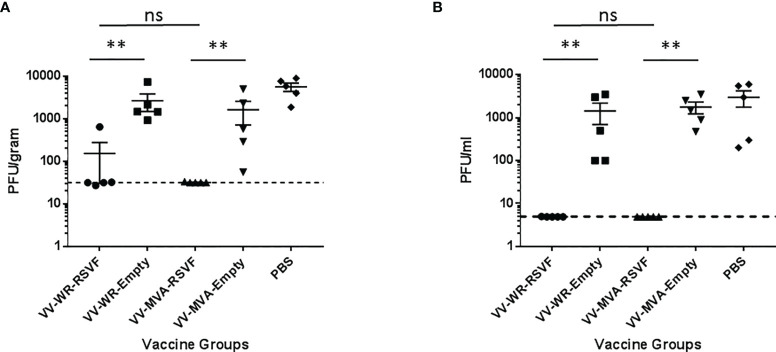
Immunization with VV-WR-RSVF augments RSV clearance in the lower and upper respiratory tract. Cotton rats were intramuscularly immunized with one dose of 10^7^ PFU of the indicated VV-WR or VV-MVA constructs and intranasally challenged with 10^6^ PFU of RSV-A2 and necropsied 5 days later with n = 5 cotton rats per treatment group. **(A)** Lung viral titre and **(B)** nose viral titre (n = 5 each) were evaluated to determine viral load in the lower and upper respiratory tract respectively. **p < 0.01 (non-parametric t-test Mann-Whitney). ns, not significant.

**Figure 6:**
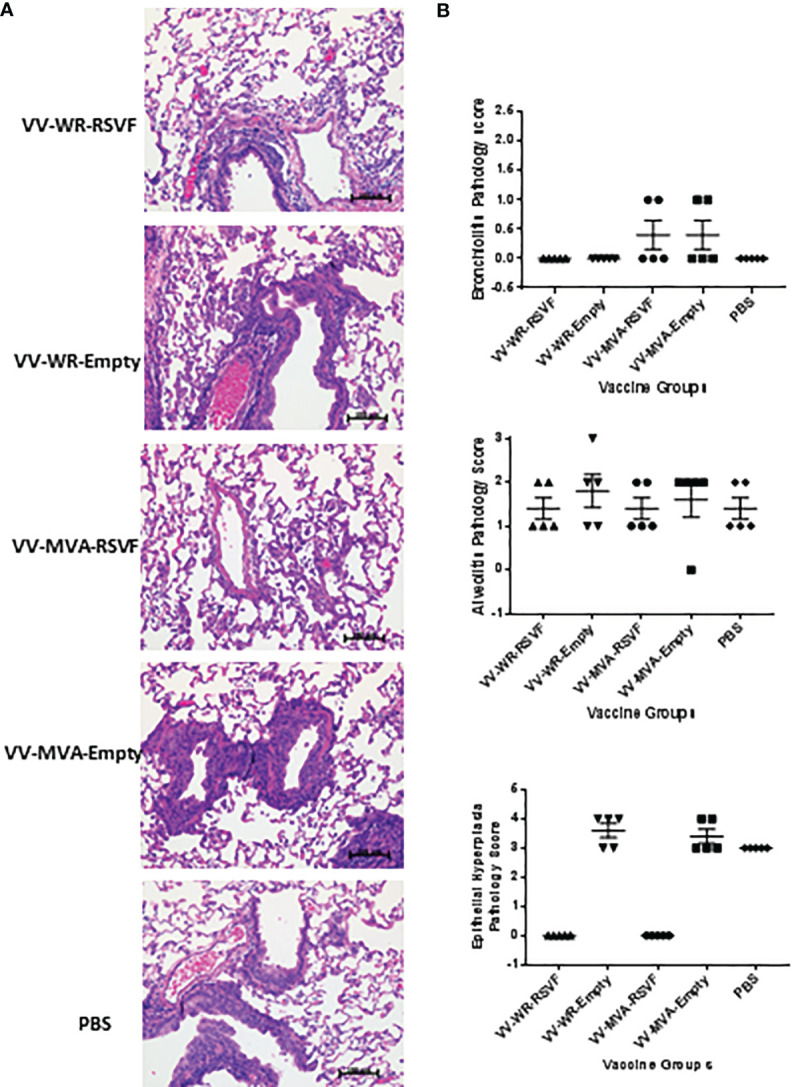
Immunization with VV-WR-RSVF does not cause ERD in cotton rats. Cotton rats were intramuscularly immunized with one dose of 10^7^ PFU of the indicated VV-WR or VV-MVA constructs and intranasally challenged with 10^6^ PFU of RSV-A2 and necropsied 5 days later with n = 5 cotton rats per treatment group. The left lobe of the lung was evaluated for histopathological changes. **(A)** Representative images of H&E stained immunized cotton rat lungs post challenge at 40x magnification. **(B)** Pathological scoring of lung tissue for bronchiolitis, alveolitis, and epithelial hyperplasia (n = 5).

RSV is known to replicate efficiently in both upper and lower respiratory tracts of cotton rats (39). We tested whether WR-RSV vaccination protected against RSV infection by evaluating lung and nasal viral loads in RSV-infected animals. Both WR-RSVF and MVA-RSVF vaccinations significantly reduced viral loads in lungs and nose when compared to PBS treated and empty vector vaccinated animals (**Figure 5**). Additionally, both vaccines reduced viral load to a similar level in RSV-infected animals. While bronchiolitis was minimal to non-existent, RSV infection did cause very mild alveolitis and moderate epithelial hyperplasia in cotton rats. Although vaccination did not have any effect on bronchiolitis and alveolitis, both WR-RSVF and MVA-RSVF were effective in completely abrogating RSV-induced epithelial hyperplasia (**Figure 6**).

## Discussion

Vaccinia, best known for its role in the vaccination campaign to eradicate smallpox, is a widely used viral vector for expression of foreign genes. Due to its excellent safety profile, MVA is the most popular VACV used for development of recombinant vaccines for several important infectious diseases, such as for AIDS, TB, Malaria and influenza (10). However, MVA has its drawbacks, as the replication incompetent vector is not as efficient as replication competent vectors in certain cases. For example, in case of a VACV-vectored rabies vaccine study, higher doses of MVA-vectored vaccines were necessary when compared to replication competent VACV-vectored vaccines in order to evoke equivalent immune responses (14). Additionally, the MVA vectored vaccines failed to induce memory responses in previously immunized animals when administered orally. Thus, further development of VACV as a safe and effective vaccine vector is warranted.

While efforts to develop a vaccine for RSV have been ongoing since the 1960s, we still do not have a safe and effective prophylactic against the disease, which is responsible for up to 22% of all severe acute lower respiratory tract infections in children (1). One of the challenges in RSV vaccine development is designing a vaccine that elicits a robust immune response accompanied with viral clearance, yet does not cause the Enhanced Respiratory Disease (ERD) first observed with Formalin-inactivated RSV vaccines (40, 41). Several advanced platforms such as protein subunit vaccines, live attenuated vaccines and vector-based vaccines have been tested and are being actively developed for RSV (42). Even with major advances in RSV vaccine research and development, there is no vaccine approved for market authorization (21, 42). Therefore, further research on vaccine platforms is necessary to develop a safe and effective vaccine against RSV.

Early studies on recombinant vaccinia for RSV vaccine development utilized the WR strain, which is the widely studied virulent laboratory vaccinia strain. These studies showed that the vaccinia vector encoding different RSV proteins were protective in various animal models such as mice, cotton rats, monkeys and chimpanzees (43–47). The WR strain of RSV is the most commonly utilized VACV research strain and shares its origin with the New York City Board of Health Strain (NYCBH), which was used in the widely deployed smallpox vaccine Dryvax ^®^ from Wyeth Laboratories (48). The WR strain was derived by repeatedly passaging the NYCBH strain first in rabbits, then in mice and more recently in diverse cell cultures. This rendered them neuropathogenic in mice and highly efficient in replicating in mammalian cultures (49). The neuropathogenic and replicating nature of the virus raised concerns regarding safety of such standard strains and hindered further development. Thus, MVA became the preferred vaccinia vector for vaccine development due to its severely attenuated nature and excellent safety profile. MVA based RSV vaccines have been tested in animal models and are protective against RSV infection (26, 50). Therefore, in this study we used the immune response elicited by the MVA-RSVF vaccine as a reference to compare the immune response elicited by our novel recombinant vaccinia based RSV vaccine.

Previously, we have developed a rapid, highly efficient and cost-effective method to construct recombinant VACV by deletion of a host range gene E3L and swap of another host range gene K3L (19). Since E3L gene is an important gene for vaccinia virus virulence and inhibition of innate immune responses, deletion of this gene would render the virus highly attenuated and more potent at inducing innate immune response. However, deletion of the E3L gene would also render the virus highly restricted in its host range, e.g. replication incompetent in most of human cells (51), thus limiting its potential application as a vector for vaccines. To improve VACVΔE3L host range, we swapped another host range gene K3L with a poxvirus ortholog TATV037. Consequently, VACVΔE3L with TATV037 are replication competent in both rodent and human cells. Therefore, we believe this platform is promising for application as a vector for vaccine development. In this study, we employed this method to construct a WR vaccinia construct expressing the RSV F gene and assessed its efficacy to protect against RSV infection in cotton rats. In order to assess the effectiveness of the WR construct we compared correlates of immunogenicity and protection with the MVA-RSVF vaccine. From our experiments, we observed that upon a single dose administration of the vaccine, WR-RSVF vaccine evokes levels of immunogenicity and protection similar to the MVA-RSVF vaccine (**Figures 4**–**6**). Additionally, we also tested the virulence of the recombinant WR backbone in mice by infecting the animals at a high dosage that would be lethal with the administration of WT WR virus. While WT WR is known to induce significant weight loss and complete lethality at an intranasal dose of 10^6^ PFU/mouse (52, 53), we did not observe such weight loss or lethality with the recombinant WR VACVΔE3L vector (**Figure 2**). WT WR virus also replicates efficiently in the respiratory tract and in the brain (31–33). Therefore, we assessed the pathology in the lungs and viral transgene expression in respiratory tract and brain after intranasal administration of the recombinant virus (**Figures 1** and **2**). We observed minimal pathology and very low transient transgene expression in the lungs. Moreover, viral transgene expression was undetectable in the brain and trachea of infected mice. This denotes that the limited replication of the VACVΔE3L vector is restricted to the lungs and the neurotropic risk associated with the WT WR virus is considerably diminished in the case of the recombinant vector that we have designed. Moreover, vaccination with WR-RSVF and subsequent challenge with RSV-A2 virus did not cause ERD as observed from the reduced lung pathological severity and viral load in WR-RSVF vaccinated and challenged animals (**Figures 5** and **6**). Thus, these observations show that the recombinant WR virus used in our study exhibits a promising safety profile for use as a vaccine vector and the protective effects of our WR-RSVF vaccine is comparable to the MVA-RSVF vaccine against RSV infection.

Some of the advantages of using this platform is that it utilizes a cost effective, highly efficient and rapid method to construct recombinant vaccinia viruses. Moreover, the usage of K3L orthologs in a ΔE3L background provides a means to adapt the recombinant viruses to be infective in specific hosts and not others (19). Therefore, another advantage of using this platform is the potential for construction of species-restricted vaccines, which may enhance the safety profile of the vaccine vector. Moreover, prime-boost vaccinations with heterologous vaccine vector backbones have been shown to be advantageous in certain situations especially when high levels of cellular immunity is needed to afford protection (54–56). Thus, the availability of a safe and effective WR vector backbone could be used in combination with other poxvirus vector backbones in heterologous prime-boost strategies to enhance immune response to certain antigens.

As peak of the incidence and severity of RSV disease occurs in infants aged <6 months in humans, it is important to vaccinate infants very early after birth. However, the Newborn immune system presents unique challenges in eliciting an effective immune response to infections and vaccinations (57), such as interference from maternal antibodies preventing generation of neutralizing antibodies and an immature immune system that is skewed towards developing a Th2 response. The inhibition of seroconversion after vaccination in the presence of maternal antibodies is not limited to RSV vaccines and has been well established for measles and pertussis vaccines (58–60). A variety of mechanisms appear to be at play in mediating maternal antibody interference, including: a) neutralization of live vaccines, b) antibody epitope masking and c) B-cell inhibition through cross-link of B-cell receptor (BCR) and FcγIIB receptor (61). However, several studies have shown that vectored and live-attenuated vaccines can be effective even in the presence of maternal antibodies and the efficacy may depend on the degree of attenuation of the vectored vaccine (62–66). Additionally, maternal antibody interference can also be overcome by employing Type I IFN stimulation and immunization with small polypeptides that are unable to cross-link BCR and FcγIIBR (67, 68). The other challenge with neonatal immunization involves the inherent immature nature of the innate immune system, which manifests as suboptimal type I and II interferon responses, reduced activation and IgG class switching of B-cells and increased Th2 and anti-inflammatory cytokine production (69). Studies showing the ability of several TLR 7/8, TLR9 adjuvants and VLPs to improve immune responses in neonatal animal models and cells demonstrate that effective adjuvants can potentially be employed to overcome the limitations of the newborn immune system (57, 69). While we tested the protective effects of WR-RSVF vaccine in adult cotton rats, the ability of the vaccine in overcoming maternal antibody interference and stimulating an effective protective response in the immature neonatal immune system is unknown. Therefore, our ongoing studies will focus on studying the ability of the WR vector and novel adjuvants in eliciting an effective immune response against RSV in infant animal models.

In summary, we constructed a VACVΔE3L based recombinant virus expressing RSVF as the antigen and TATV037 as the selection marker enabling the virus replication in both human and rodent cells. As a proof of concept, the novel platform used in this study is comparable to MVA with respect to the immunogenicity profile and protection. Our preliminary safety study suggests that the virulence of the vector has been substantially abolished compared with the WT WR virus. Nonetheless, systematic evaluation of long-term protection and safety is still needed, which is ongoing in our laboratories. Moreover, while we have conducted a comprehensive analyses of safety and efficacy of the vector, more mechanistic studies could be considered to include systematic analyses of cytokines profiles and immune cell phenotypes, which is understandably a challenge, given the scarcity of the reagents for cotton rats.

## Data Availability Statement

The raw data supporting the conclusions of this article will be made available by the authors, without undue reservation.

## Ethics Statement

The animal study was reviewed and approved by Health Canada’s Institutional Animal Health Care Committee.

## Author Contributions

MR, ST, WC, GD, DS, MJ, SS, LW, MR-M, JC, and XL were involved in conceptualization of the project. MR, ST, CG, WZ, and AP were involved in conducting the experiments and generation of data. MR, ST, CG, WZ, AP, WC, GD, DS, MJ, SS, LW, MR-M, JC, and XL were in involved in data interpretation and writing of the manuscript. WC, GD, DS, MJ, SS, MR-M, and XL were responsible for the acquisition of the research funding. All authors contributed to the article and approved the submitted version.

## Funding

The work is funded by Genomics Research and Development Initiative from the Government of Canada.

## Conflict of Interest

The authors declare that the research was conducted in the absence of any commercial or financial relationships that could be construed as a potential conflict of interest.

## Publisher’s Note

All claims expressed in this article are solely those of the authors and do not necessarily represent those of their affiliated organizations, or those of the publisher, the editors and the reviewers. Any product that may be evaluated in this article, or claim that may be made by its manufacturer, is not guaranteed or endorsed by the publisher.
